# A New Design of Porosity Gradient Ti-6Al-4V Encapsulated Hydroxyapatite Dual Materials Composite Scaffold for Bone Defects

**DOI:** 10.3390/mi12111294

**Published:** 2021-10-21

**Authors:** Cheng-Tang Pan, Wen-Hsin Hsu, Yu-Shun Cheng, Zhi-Hong Wen, Wen-Fan Chen

**Affiliations:** 1 Department of Mechanical and Electro-Mechanical Engineering, National Sun Yat-Sen University, Kaohsiung 80424, Taiwan; pan@mem.nsysu.edu.tw (C.-T.P.); sheng1102@mem.nsysu.edu.tw (Y.-S.C.); 2 Department of Emergency Medicine, Kaohsiung Armed Forces General Hospital, Kaohsiung 80284, Taiwan; acidosis1976@yahoo.com.tw; 3 Institute of Medical Science and Technology, National Sun Yat-Sen University, Kaohsiung 80424, Taiwan; 4 Department of Marine Biotechnology and Resources, National Sun Yat-Sen University, Kaohsiung 80424, Taiwan; wzh@mail.nsysu.edu.tw

**Keywords:** critical bone defect, composite scaffold, porosity, Ti-6Al-4V, hydroxyapatite, finite element analysis, stress shielding effect

## Abstract

The tibia of New Zealand White rabbits was used as a model of critical bone defects to investigate a new design of composite scaffold for bone defects composed of dual materials. The all-in-one design of a titanium alloy (Ti-6Al-4V) scaffold comprised the structure of a bone plate and gradient porosity cage. Hydroxyapatite (HAp), a biodegradable material, was encapsulated in the center of the scaffold. The gradient pore structure was designed with 70%-65%-60%-55%-50% porosity, since the stresses could be distributed more uniformly when the all-in-one scaffold was placed on the bone contact surface. By covering the center of the scaffold with a low strength of HAp to contact the relatively low strength of bone marrow tissues, the excessive stiffness of the Ti-6Al-4V can be effectively reduced and further diminish the incidence of the stress shielding effect. The simulation results show that the optimized composite scaffold for the 3D model of tibia had a maximum stress value of 27.862 MPa and a maximum strain of 0.065%. The scaffold prepared by selective laser melting was annealed and found that the Young’s coefficient increased from 126.44 GPa to 131.46 GPa, the hardness increased from 3.9 GPa to 4.12 GPa, and the strain decreased from 2.27% to 1.13%. The result demonstrates that the removal of residual stress can lead to a more stable structural strength, which can be used as a reference for the design of future clinical tibial defect repair scaffolds.

## 1. Introduction

Currently, the regenerative healing of bone diseases has been the subject of many studies in clinical medicine. From the aspect of bone cell growth, Bobyn et al. [1] in 1980 demonstrated that the optimal growth pore size for stainless steel porous implants should be in the range of 50–400 μm. In 2016, Naoya Taniguchi et al. [2] proposed that the porous bone replacement made of titanium alloy has the best implant fixation ability and growth efficiency at a pore size of 600 μm. In 1986, Schmitz and Hollinger defined the term “Critical-Sized Defect (CSD)” as a condition in which a bone defect is incapable of repairing itself [3], which is often caused by massive trauma, skeletal disease, tumor, and abnormal bone growth [4].

For the healing of large defects, the use of bone grafting is currently the most common treatment, which includes autograft, allograft, and artificial bone graft substitute material [5]. In the clinical study, Arrington et al. [6] in 1996 used autologous and allogeneic bone grafting to fill defects and induce bone tissue regeneration. However, the study found that post-implantation complications such as rejection and infection may occur with autologous transplants. In 2001, Moore et al. [7] showed that the sterility of artificial bone graft was effective in reducing the incidence of complications. In 2007, Vecchio et al. [8] converted shell components to hydroxyapatite (HAp) as artificial bone, which showed superior biocompatibility and bioactivity. HAp is a highly biocompatible material with degradable and absorbable properties, which is considered to be one of the commonly used biomaterials. However, due to its inherent brittle properties, it can only be used under relatively low loading stresses [9]. Recently, in 2019, Zhang et al. [10] showed that the use of titanium alloys in artificial bone can provide better support during the healing process and effectively reduce the occurrence of infections and other problems.

Nowadays, there are many artificial bone designs using Ti-6Al-4V as a material, which has better biocompatibility and corrosion resistance than other metal alloys [11]. However, in terms of material strength, the Young’s modulus of Ti-6Al-4V is about 90–115 GPa, which is much higher than that of cortical bone (17 GPa [12]) and cancellous bone (5 GPa [13]). Consequently, the uneven distribution of stresses in titanium alloy artificial bone after implantation would cause excessive stress concentration, which leads to a stress shielding effect. This results in reduced regeneration of the bone tissue, leading to the risk of slippage and collapse of the implant during the recovery period [14]. Therefore, many researchers are devoted to investigating different structural designs and combinations to overcome the steel properties of Ti-6Al-4V materials to achieve superior bone tissue growth [15,16,17]. In 2018, Marie Pobloth et al. [18] combined the designed implant with a fixation plate, which was shown to be effective in increasing the stability of the structure after implantation. In 2020, Gurpreet et al. [19] combined Ti-6Al-4V and HAp materials in the implant design, so that it had the high strength of titanium alloy and also the high biocompatibility of HAp, in order to greatly enhance the viability of the implant.

In the past, most of the titanium replacements were manufactured using traditional processing methods, and the design tended to be simpler in structure, resulting in the metal replacements only being superficially wrapped by the tissues after implantation. The development of selective laser melting (SLM) in recent years has made it possible to successfully process metallic materials using the additive manufacturing method. In 2017, Michaela et al. [20] successfully processed Ti64 powder material by SLM technology to complete the design of porous bone replacements, and the results show that the fabricated replacements maintained good biocompatibility after implantation.

Although SLM technology is a fast and efficient way to process porous structure designs, there are still some problems, such as a large amount of stress residues. The main reason for this is that the temperature gradient of this technique varies too much in a short period of time during the manufacturing process, and the residual stress reduces the strength of the material itself, causing problems such as cracks during stressing [21]. According to a technical report provided by NASA STI Repository (NTRS) in 1966, the best stress removal conditions for Ti64 material are set at 537 °C to 648 °C, and the heating time is controlled at 15 to 60 min, followed by natural cooling to ensure that the material is cooled down without significant changes in properties. This ensures the most effective residual stress removal without significant changes in the properties of the material [22]. In 2019, Ettefagh et al. [23] processed the Ti-6Al-4V material by SLM technique and then annealed it, and found that the corrosion resistance and structural strength were improved after annealing treatment [23].

The study proposed a regenerative composite scaffold for tibial defective tissues. By combining the advantages of Ti-6Al-4V and HAp materials, the gradient pore cage with bone plate was designed to promote cell regeneration by tuning the pore size of the porous structure. The gradient support structure with 50–70% porosity was used to control the stress distribution problem generated by the bone plate. The structure design was expected to achieve the effect of bone plate support and fixation, and at the same time, not cause the uneven distribution of force on the implant. The design of the implant was verified by the finite element analysis (FEA) method to optimize the structure.

## 2. Methods

### 2.1. Experimental Model

**Figure 1:**
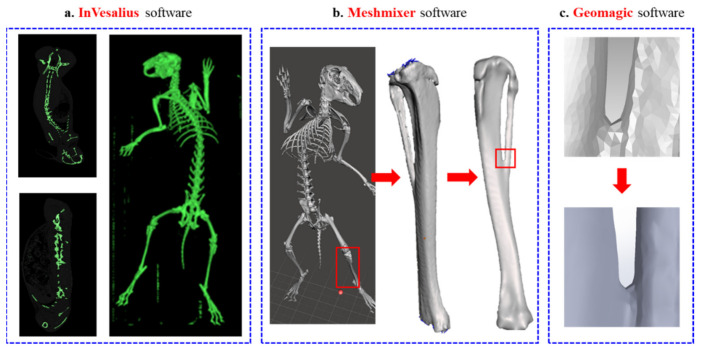
(**a**) InVesalius software, (**b**) Meshmixer software, and (**c**) Geomagic software.

In this study, the tibia of the left leg of an adult New Zealand White rabbit was selected as the target site, and a critical bone defect size of 5 mm was designed in the middle part of the tibia. To preserve the maximum fidelity for FEA, the rabbit images were scanned by computed tomography (CT) and imported into the medical overlay modeling software InVesalius 3.1 (CTI Renato Archer, Brazil) by digital imaging and communications in medicine (DICOM) format. Figure 1 shows the process of tibial defect modeling. The initial 3D model of the tibial tissue was captured and reconstructed by adjusting the grayscale contour of the image to the same scale size, as shown in Figure 1a. The rough skeletal model was repaired by surface repair software Meshmixer (Autodesk Inc, San Rafael, CA, USA) and reverse engineering software Geomagic Studio (3D system Inc, Rock Hill, SC, USA) for surface roundness and damage repair, as shown in Figure 1b. A high fitting degree of surface mesh structure was cut, as shown in Figure 1c, to convert the skeletal 3D model into an international standard file format (STP) that can be edited by computer-aided design (CAD) drawing software.

In the construction of the defect model, preliminary dimensional measurements were made by graphic software, and the total length of the tibia was ~105 mm. A 2.5 mm horizontal symmetrical defect incision was made at the midpoint of the model, and the internal structure of the model was divided into the cortical bone, the reserved bone marrow and cancellous bone. The purpose of this approach is to differentiate the internal tissues of the tibia by different material mechanics when using FEA. The use of this method to model the critical defect of tibial supports in the design of a bone defect scaffold with realistic tibial dimensions, and the model was used as an environmental field for mechanical FEA to increase the authenticity and reliability of the overall study.

### 2.2. Design of Scaffold for Bone Defect

**Figure 2:**
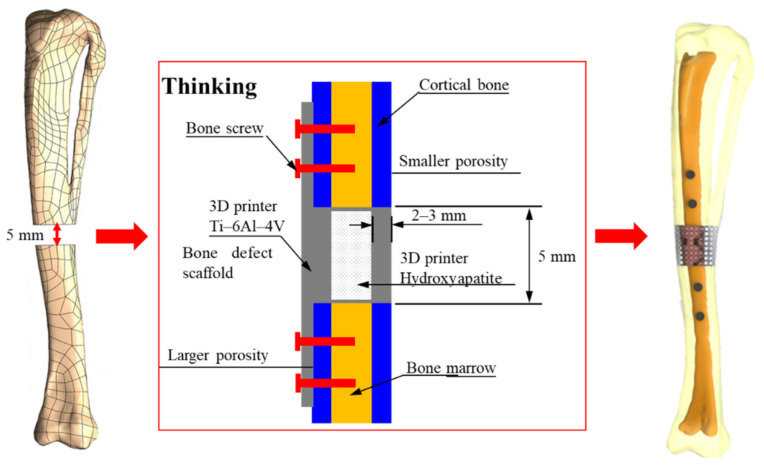
Design of titanium alloy (Ti-6Al-4V) material as a framework, covered with hydroxyapatite (HAp) material.

Based on the current medical technology, titanium alloy material has been widely used in the design of invasive implants for bone regeneration, and it is also the primary choice of material for many medical devices at present. Compared to other alloys, Ti-6Al-4V has excellent biocompatibility and resistance to wear and corrosion. However, the material strength of titanium alloy is higher than the Young’s modulus of cortical bone tissue (17 GPa [12]), which may cause a stress shielding effect of the replacement after implantation. In this study, the Ti-6Al-4V material was used as a framework to cover the HAp material, as shown in Figure 2. The design would protect the fragility of HAp during the initial stage of implantation and also induce bone regeneration with high biocompatibility and biodegradable properties to achieve an excellent bone repair effect.

In this study, two material structures were fabricated by different 3D printing methods. The main Ti-6Al-4V material structure was processed by selective laser melting (SLM), while the HAp material structure was processed by additive manufacturing (AM). No adhesive was used for the bonding of the two materials in order to avoid additional adverse biological reactions after implantation into the body. The method was based on the structural interlocking of the two materials through their own design dimensional differences.

**Figure 3:**
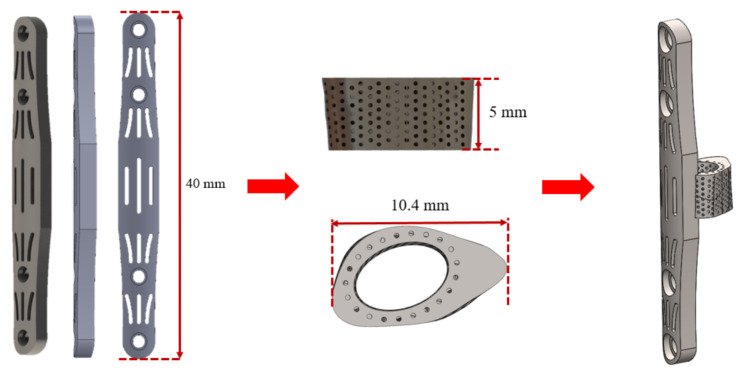
Regenerative replacement body (cage) and bone plate one-piece molding design.

Bone plates are commonly used in medical orthopedics for the treatment of fractures and for internal fixation after surgery to prevent extensive displacement of the bone during recovery [24]. In the study, the regenerative replacement body (cage) and the bone plate were designed to be integrated into one piece, as shown in Figure 3. The design can effectively stabilize the cage during the recovery period (the tissue has not fully grown into the replacement body) and also can reduce the risk of implant slippage due to external forces.

**Figure 4:**
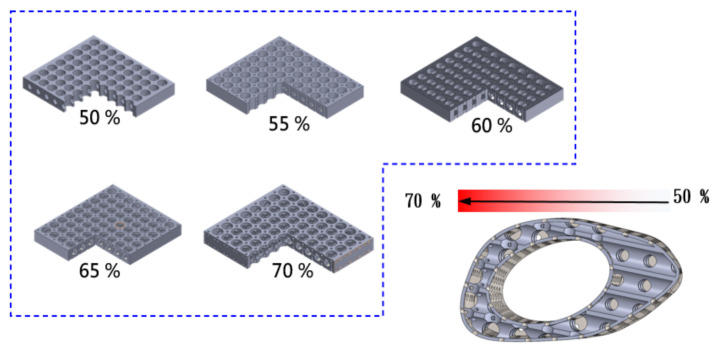
Gradient porosity arrangement as a control method, from 70%-65%-60%-55%-50%, divided into five layers of different structural arrangements.

The pore design was based on the optimal regeneration of bone tissue, with an average pore size of 50 to 600 μm. From the mechanical point of view, the gradient porosity arrangement was used as the control method, which was divided into five layers (70%-65%-60%-55%-50%) to reduce the stress concentration problem, as shown in Figure 4. Because of the stronger support near the bone plate, the force that can be carried is relatively large, which results in the problem of the uneven distribution of force. By controlling the gradient porosity, the problem can be effectively improved, and the overall regenerative composite scaffold structure can also retain the advantages of high stability and the optimal regeneration conditions of the composite scaffold.

### 2.3. Finite Element Analysis

**Figure 5:**
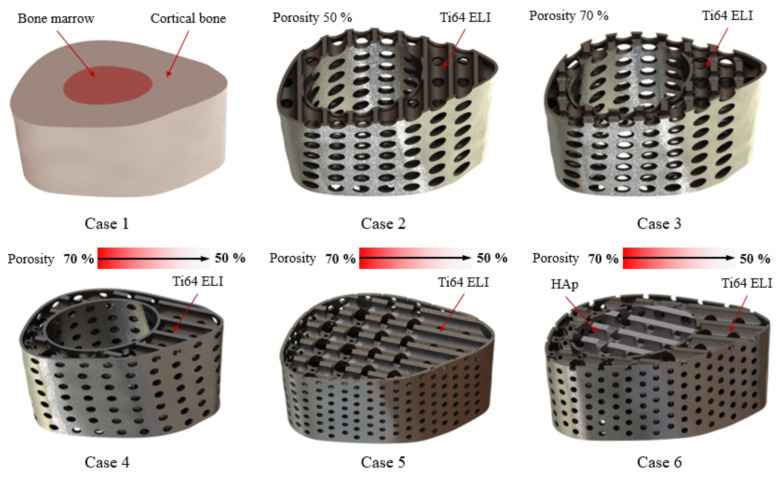
Experimental design for FEA.

Finite element analysis (FEA) was performed by the computer-aided simulation software Ansys-Workbench (Software Corporation, Canonsburg, PA, USA) to analyze the mechanics of the designed composite scaffold for tibial defects occurring in the real rabbit environment. Dual materials (Ti-6Al-4V and HAp) and various porosity combinations were used to obtain the optimal design conditions for the designed structure to achieve the minimum stress concentration. The experimental designs are shown in Figure 5 and Table 1. The results of the equivalent stress simulations were used to further optimize the design of the structure by matching the structural strength requirements and damage levels.

For the mechanical analysis, the skeletal structure materials, Ti-6Al-4V, and HAp materials were used for the parameter design. The skeletal structure of the tibia was differentiated into different values for cortical bone, cancellous bone, and bone marrow, which include Young’s modulus (Ε), Poisson’s ratio (ν), density (ρ), and yield strength (σ_yield_), as shown in Table 2.

**Figure 6:**
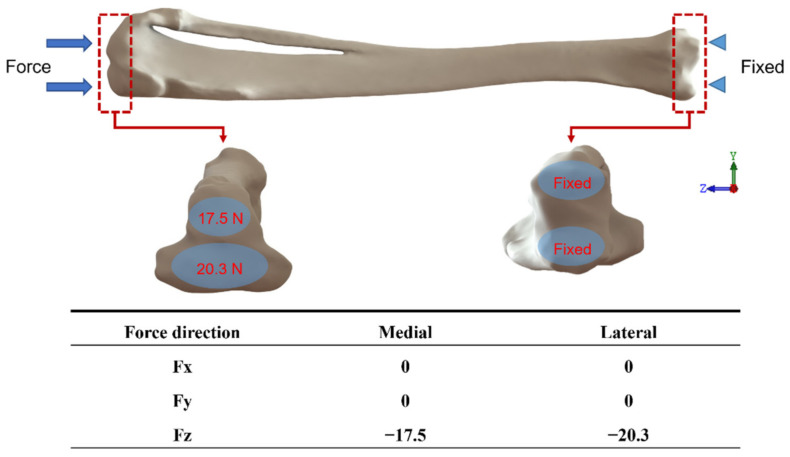
The boundary setting is based on the joint force of 17.5 N on the inner side and 20.3 N on the outer side of the axis as the main force.

For the design of the boundary conditions, the magnitude and direction of force on the tibia at rest were used to set up the model, where the loading was only set in the same direction as the *Z*-axis. The reason for this was to simulate the loading conditions on the tibia of the rabbit under normal static conditions and to define the detailed positions and magnitudes of the applied forces. The location of each force was estimated based on the study of Gushue et al. [28] and the force magnitude was designed according to the study of Perez et al. [29]. The main force was 17.5 N on the medial side of the axis and 20.3 N on the lateral side of the joint, without considering the force effects of the muscles and ligament tissues, and was fixed at two points on the lower end of the tibia, which are shown in Figure 6.

### 2.4. Ti-6Al-4V Powder Melt Processing and Stress Relief Annealing

In this study, the CAD drawing files of the scaffold design were transferred to an STL coordinate data file, and the test sample was manufactured by the SLM machine (EOSINT M280). The process used an optical fiber laser to melt the Ti6-Al-4V powder (EOS, Krailling, Germany), which was dedicated to the EOSINT M system, and then melted and molded the scaffolds layer by layer according to the design. The prepared samples were subjected to a stress relief annealing process with the parameters shown in Table 3.

### 2.5. Material Properties Measurement

**Figure 7:**
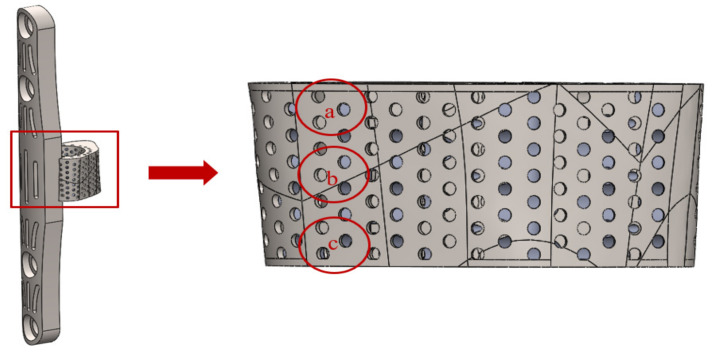
Pore size measurement of titanium cage.

The pore size of the Ti-6Al-4V specimen was measured with an optical microscope (OM) at an objective magnification of X5.0, and the surface morphology was observed with a scanning electron microscope (SEM). The measurement area was defined as the pore on the right side of the titanium cage surface near the bone plate, and the three areas of a, b, and c were listed in the axial direction, as shown in Figure 7.

**Figure 8:**
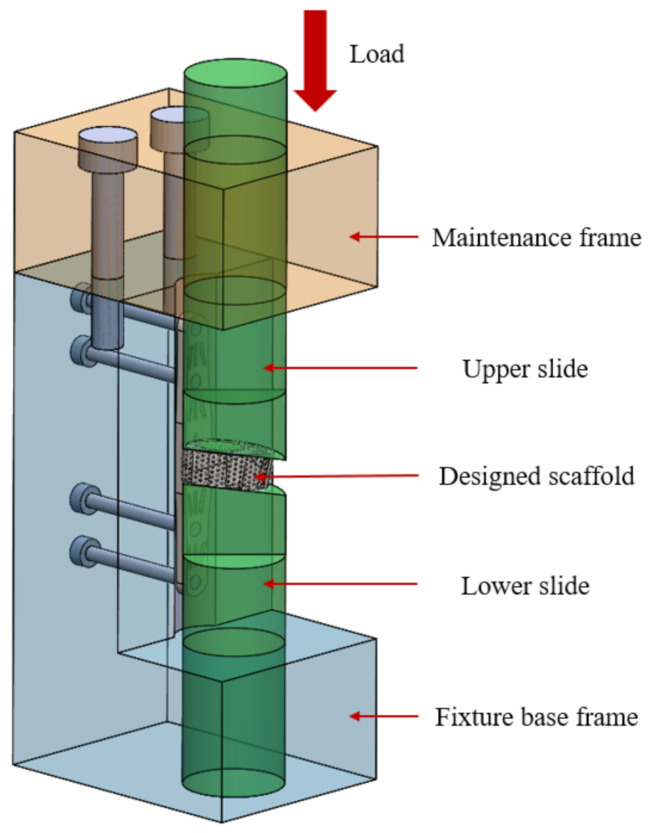
Fixture design architecture.

The Nano Indenter XP System (MTS Systems Corporation, Eden Prairie, MN, USA) was used to measure the mechanical properties of the scaffolds after annealing treatment. The system’s built-in continuous stiffness measurement (CSM) was used to indent the surface of the sample in a 9-point array before and after annealing by grinding and removing the oxide layer. The distance between points was set to 5000 nm, the indentation depth was set to 2000 nm, and the needle was placed at a frequency of 45 Hz and a rate of 10 nm/s. The compressive strength and fatigue strength of the samples were measured by the electronic universal testing machine AGS-X (SHIMADZU, Kyoto, Japan). The designed fixture was mainly composed of four modules, which had an L-shaped fixture base, lower track maintenance frame, and upper slider and lower slider; its assembly structure is shown in Figure 8. The fixture was manufactured by traditional metal processing technology using high-strength stainless steel material. Before conducting the test of compressive strength and the fatigue test of the universal testing machine, in order to avoid the difference effect caused by the surface fitting, the top and bottom surfaces of the specimen were ground by water sandpaper with a roughness of #50~#2000.

The compressive strength test was used to investigate the displacement and strain generated by the scaffold under quantitative loading. Three sets of unannealed and annealed specimens were taken and installed in the designed fixture. The single-direction control mode of the universal testing machine was used, and the maximum value of 150 N was set at three times the weight of an adult rabbit. The test was completed by a continuous load of 0–150 N and a load rise speed of 0.001 kN/s. The fatigue strength test was used to verify that the designed scaffold could maintain a limited amount of small deformation under cyclic loading. The experiment was carried out in step control mode with three sets of specimens after annealing, and 10,000 cycles were completed at a frequency of 0.5 Hz between 50 and 150 N.

## 3. Results and Discussion

A 3D model of a critical bone defect of 5 mm in size was created and a porosity-gradient composite scaffold was designed based on the range of the defect. A FEA was used to simulate the mechanical properties of the design under the implantation environment to compare the autologous bone graft, single-porosity Ti-6Al-4V scaffold, and porosity-gradient Ti-6Al-4V scaffold in the same condition.

### 3.1. Autologous Bone Graft

**Figure 9:**
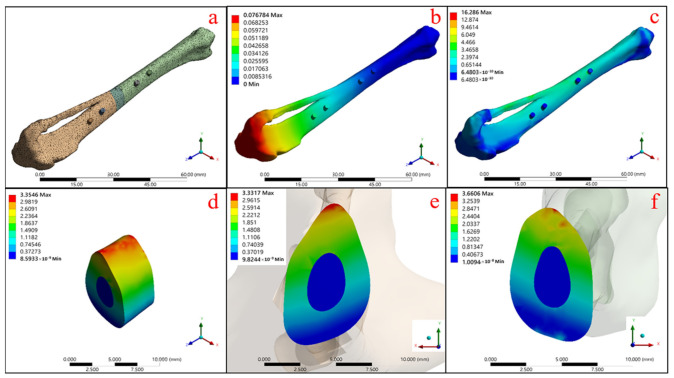
Autologous bone graft state: (**a**) the model and mesh building, (**b**) the total deformation of the overall mode and the equivalent stress, (**c**) the overall model, (**d**) the grafting bone, (**e**) the upper contact surface, and (**f**) the bottom contact surface.

An autologous bone model with the same material properties as the original bone was used as the setting value. The post-operative fixation was performed with the addition of a split bone plate and studs to simulate the mechanical performance of the autologous bone graft, as shown in Figure 9 and Table 4.

Figure 9a shows the overall model with a grid segmentation condition for the finite elements of mechanics, and Figure 9b confirms that the maximum total deformation of the model under the set boundary force condition is 0.077 mm. Figure 9c shows the stress distribution of the overall model with the maximum stress concentration of 16.286 MPa, and then the range of this model reduced to the autologous bone block (shown in Figure 9d), which reveals that the maximum stress on the bone block is 3.355 MPa. Figure 9e,f show the maximum stress distribution between the upper and lower contact surfaces of the tibia model at the defect location with the graft, respectively, with 3.332 MPa for the upper contact surface and 3.661 MPa for the lower contact surface.

### 3.2. Single-Porosity and Porosity-Gradient Ti-6Al-4V Scaffolds

**Figure 10:**
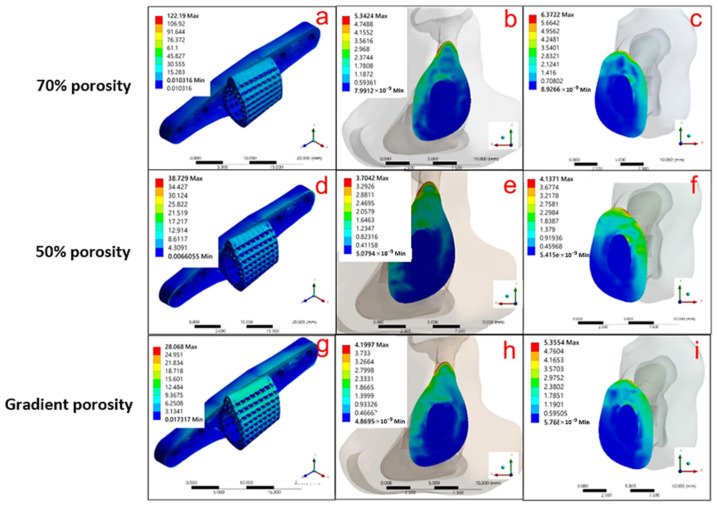
Three different angles, including scaffold body, upper contact surface, and lower contact surface, were used to view the stress distribution in the scaffold composed of different structures: (**a**–**c**) 70 % single porosity, (**d**–**f**) 50 % single porosity, and (**g**–**i**) pore gradient.

In this study, the bone plate was attached to the bone surface in a free-positioned manner in the model, and was locked in place by setting boundary conditions on the bone studs in the FEA simulation as a fixation method. The Ti-6Al-4V scaffold was structurally arranged with gradient porosity to control the stress concentration problem arising from the contact surface between the scaffold and the bone tissue, which also compared with the simulated results of a single porosity of 70% and 50%. The simulated stress distribution and the equivalent stress and strain parameters are shown in Figure 10 and Table 5, respectively.

Figure 10a–c show the result of the force simulation of the single-porosity 70% structured scaffold, which indicates that the maximum stress is 122.19 MPa for the Ti-6Al-4V scaffold (Figure 10a), 5.342 MPa for the contact surface above the tibia (Figure 10b), and 6.372 MPa for the contact surface below the tibia (Figure 10c). The simulation results for the single-porosity 50% structured scaffold are shown in Figure 10d–f, with 38.729 MPa for the Ti-6Al-4V scaffold (Figure 10d), 3.704 MPa for the contact surface above the tibia (Figure 10e), and 4.137 MPa for the contact surface below the tibia (Figure 10f). Hence, the results of simulations with different single-porosity structures suggest that the porosity can affect the force performance of the scaffold under the implantation environment. Furthermore, the simulation performed with the gradient-porosity scaffold structure is shown in Figure 10g–i. The results demonstrate that the stress for the Ti-6Al-4V scaffold was 28.068 MPa (Figure 10g), 4.2 MPa for the upper contact surface of the tibia (Figure 10h), and 5.355 MPa for the lower contact surface (Figure 10i). The results clearly indicate that the gradient porosity structure has superior stress distribution, which can retain the structural strength of the scaffold under the implantation environment without causing stress compression of the original bone tissue due to stiffness problems.

### 3.3. Ti-6Al-4V Combined with HAp Material for Pore Gradient Composite Scaffold Design

**Figure 11:**
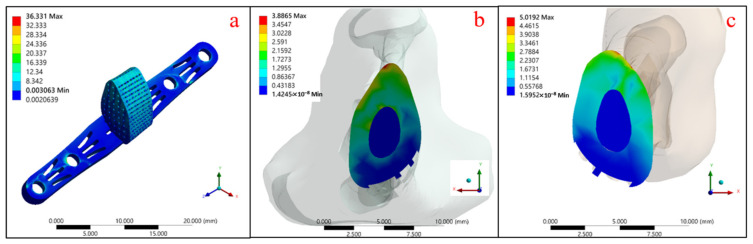
Stress distribution of single Ti-6Al-4V material pore gradient support: (**a**) Ti-6Al-4V support body, (**b**) upper contact surface, and (**c**) lower contact surface.

The gradient-porosity scaffold composed of a single Ti-6Al-4V metal was analyzed by the mechanical FEA method, as shown in Figure 11 and Table 6. It can be seen that the maximum stress of the stent structure was 36.331 MPa (Figure 11a), with 3.887 MPa at the upper contact surface (Figure 11b) and 5.019 MPa at the lower contact surface of the tibial defect (Figure 11c).

**Figure 12:**
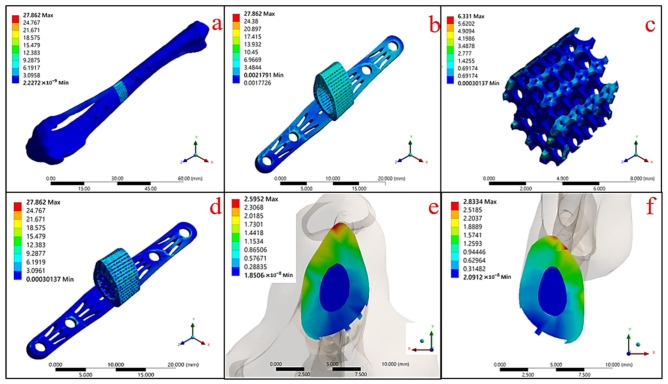
Mechanical simulation results of Ti-6Al-4V and HAp gradient pore scaffolds in a biological environment: (**a**) overall skeletal implantation environment, (**b**) Ti-6Al-4V metal scaffold structure, (**c**) HAp material structure, (**d**) bimaterial structure, (**e**) upper contact surface, and (**f**) lower contact surface.

The results of the mechanical simulation of the gradient porosity composite scaffold structure composed of dual materials are shown in Figure 12 and Table 7. It can be seen that the maximum stress of the structural implant under the tibial model was 27.862 MPa (Figure 12a), in which the stress was concentrated mainly on the outer frame of the Ti-6Al-4V metal scaffold (Figure 12b). Furthermore, the stress of the HAp structure was 6.331 MPa (Figure 12c).

From the above simulation results, it can be seen that the overall scaffold and the upper and lower contact surfaces of the single Ti-6Al-4V stent structure were subject to greater stress concentration compared with the dual-material composite scaffold due to the high stiffness of the material. Hence, the addition of HAp can significantly improve the effect, and also achieve the purpose of protecting the fragile HAp material with a high-strength Ti-6Al-4V material structure.

**Figure 13:**
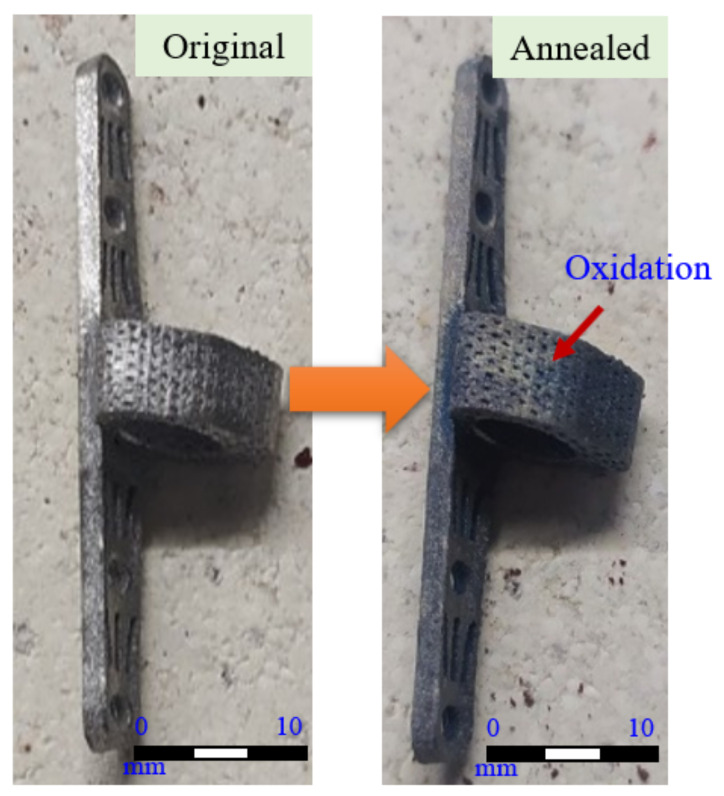
Surface condition of the specimen before and after stress relief annealing treatment.

The titanium alloy specimens processed by the melt process were found to have obvious oxidation on the surface after stress relief annealing treatment, as shown in Figure 13. According to Bruni et al. [30], the formation of a Ti-6Al-4V oxide layer does not adversely affect biocompatibility, but rather provides resistance to wear and tear.

**Figure 14:**
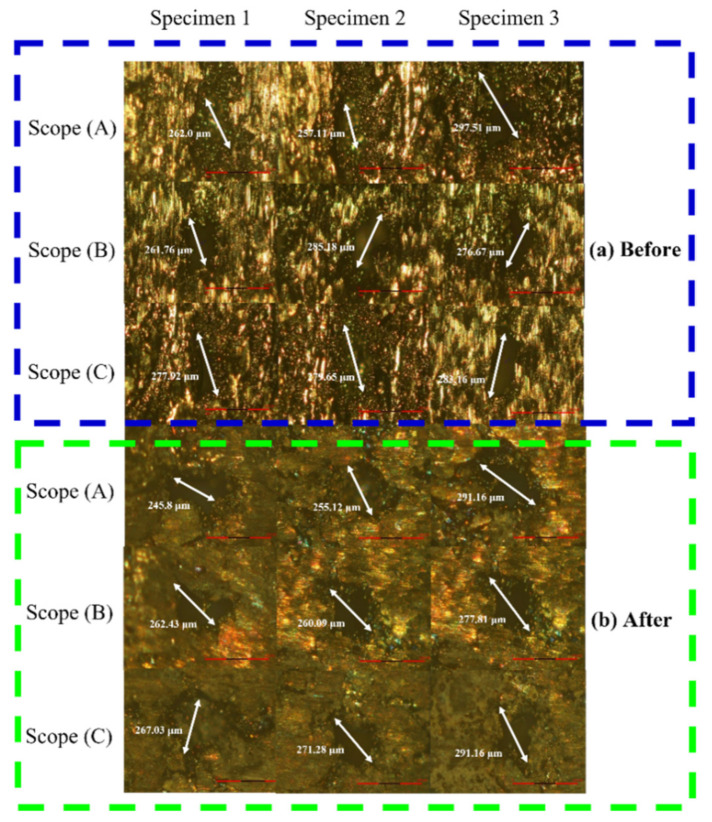
OM observation results of surface pores of (**a**) initial and (**b**) annealed specimens.

The results of measuring the pore size on the surface of the specimen by OM measurement before and after stress relief annealing treatment and the fatigue test are shown in Figure 14, and the data are compiled as shown in Table 8. It was found that the specimens did not cause extensive deformation of the surface pores after annealing treatment and cyclic loading.

**Figure 15:**
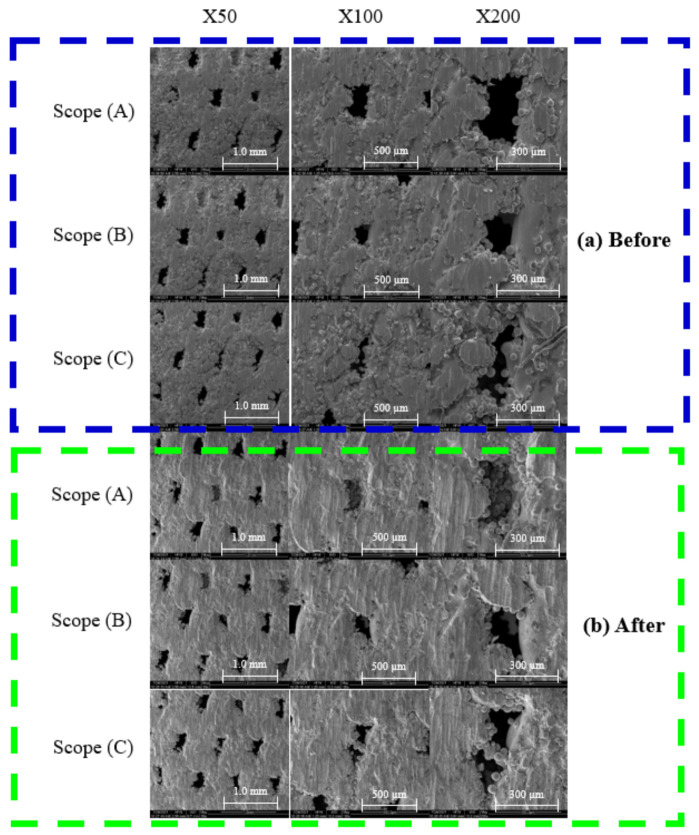
SEM images of surface pores of (**a**) initial and (**b**) annealed specimens.

A comparison of the initial molding effect on the surface of the specimen with the specimen after stress annealing and the fatigue test showed that there are still many defects on the surface without stress relief annealing, including surface cracks and incomplete melting of the metal powder particles, as shown in Figure 15a. However, after the annealing treatment, there was a significant improvement in the defects, as shown in Figure 15b.

**Figure 16:**
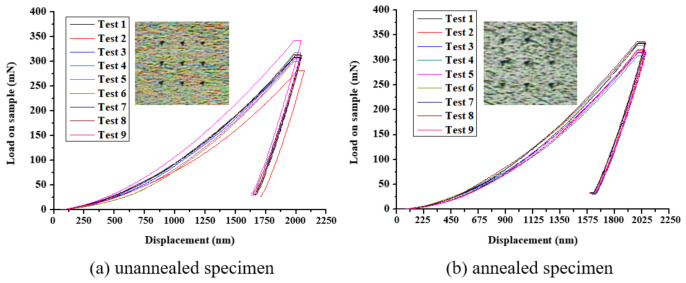
Load–displacement relationship curve of an (**a**) unannealed and (**b**) annealed specimen.

Figure 16 shows the load–displacement (L–D) relationship curves of the nine-point array of the titanium alloy specimens before and after annealing treatment at a depth of 2000 nm, measured by the nanoindentation system. The results show that the average Young’s coefficient for the original specimen before annealing was 126.44 GPa and the average hardness value was 3.9 GPa, as shown in Figure 16a. However, these values are lower than those of the annealed specimens, as shown in Figure 16b, with an average Young’s coefficient of 131.46 GPa and an average hardness value of 4.12 GPa, which confirmed that the average Young’s modulus and the average hardness could be increased after annealing treatment due to the removal of residual stress [22,26]. A comparison of the data is shown in Table 9.

**Figure 17:**
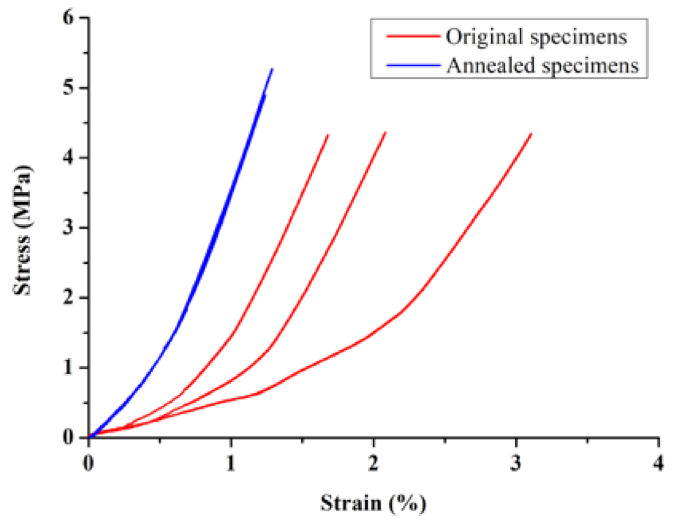
Stress–strain relationship curves before and after annealing treatment of the sample.

The results of the compressive strength of the specimen (S–S relationship curve) are shown in Figure 17. When the unannealed specimens were loaded to 50 N, 100 N, and 150 N, the overall average strain was 1.42%, 1.89%, and 2.27%, and the average strain of the annealed specimens was reduced to 0.58%, 0.89%, and 1.13%, respectively. The detailed results are shown in Table 10. The results show that the annealed specimens produced lower strain under the same load, and the three highly fitted stress–strain relationship curves demonstrate the trend of a more stable overall structure.

**Figure 18:**
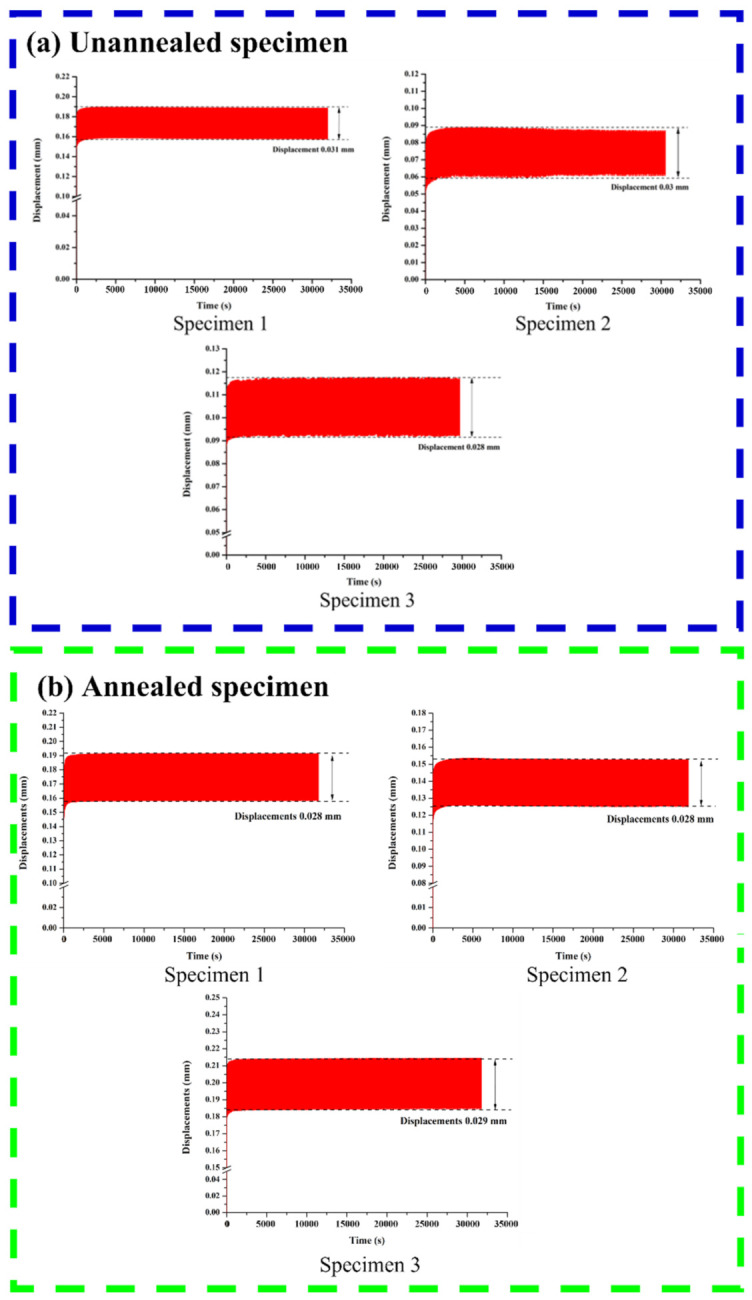
Displacement–time waveform curves for an (**a**) unannealed and (**b**) annealed specimen.

Displacement–time waveform curves for unannealed and annealed specimens are shown in Figure 18. The fatigue test results of the unannealed specimen show that the displacement interval was 0.03 mm on average, under cyclic loading between 50 N and 150 N, while that of the annealed specimen was 0.028 mm on average, indicating that the displacement–time waveform curve did not shift significantly due to the strength properties of the Ti-6Al-4V material, so the structure was not damaged significantly during the process.

### 3.4. Discussion

Based on the above simulation results, when the tibia model was subjected to axial forces from two joint contacts, the different materials and structures of the replacements significantly affect the distribution and magnitude of the equivalent stresses. The maximum equivalent stress values for the different designs of the replacement models are shown in Figure 10. The maximum stress generated by the autologous bone graft method was 3.355 MPa. Further, the stresses were 122.19 MPa and 38.729 MPa, respectively, when the scaffold portion was designed with a 70% and 50% single-porosity structure, while the stress was 28.068 MPa when the gradient porosity design was applied. The results indicate that the control of porosity can effectively improve the problem of stress concentration in the replacement body.

**Figure 19:**
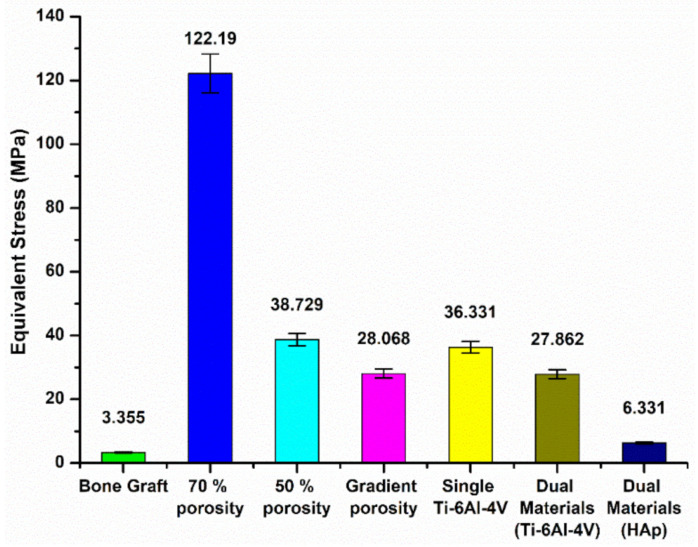
Maximum equivalent stress value of each replacement model material.

Figure 19 compares the simulation results for the single Ti-6Al-4V scaffold with the dual-material composite scaffold. The maximum stress on the single Ti-6Al-4V structure was 36.331 MPa, while the maximum stress on the Ti-6Al-4V support was 27.862 MPa when the center of the support was covered with HAp. Only 6.331 MPa was concentrated on the HAp material. Hence, it can be seen that the dual-material composite scaffold design can meet the condition of uniform force distribution, and also achieve the effect of protecting the HAp material by using the Ti-6Al-4V material as support.

**Figure 20:**
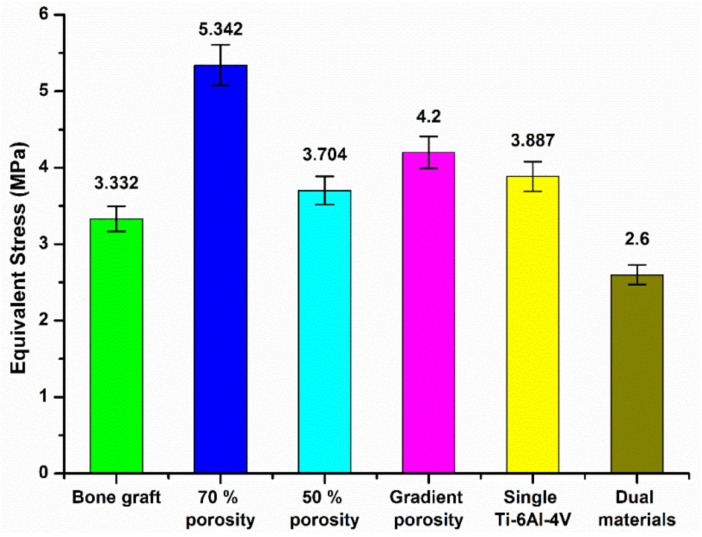
Equivalent stress values of the contact surface between the tibial defect site and the upper endpoint of the replacement.

**Figure 21:**
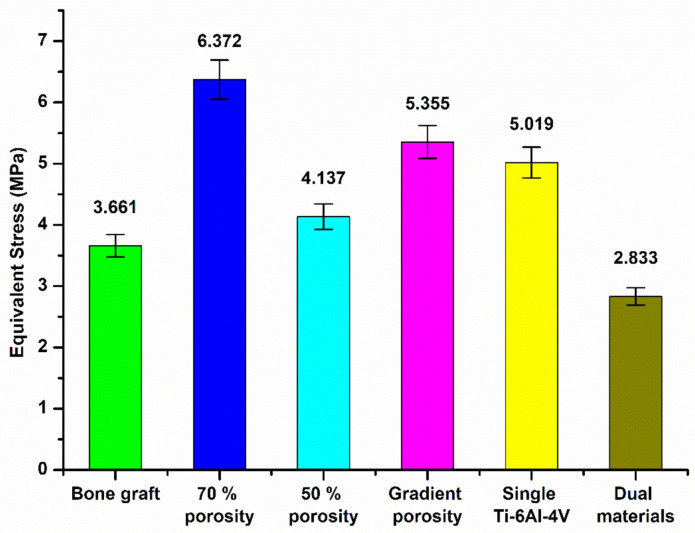
Equivalent stress values between the tibial defect site and the contact surface of the lower end of the replacement body.

Figure 20 and Figure 21 are the equivalent stress values between the tibial defect site and the contact surface of the upper and lower end points of the replacement body, respectively. In the case of the autologous bone replacement body, the maximum stresses of 3.332 MPa and 3.661 MPa were caused at the upper and lower end contact surfaces, respectively. However, the dual-material design of the composite scaffold caused maximum stresses of 2.6 MPa and 2.833 MPa at the upper and lower end contact surfaces, respectively, demonstrating that the design was effective in reducing the skeletal injuries caused by the stiffness of the material. Further, the stress performance of the final design is close to the real skeleton, which can also reduce the incidence of the stress shielding effect.

Based on the results of the actual measurements on the specimens processed by SLM, it was found that this technology can be applied to the processing of complex structural scaffolds at the micron level, the microscopic pores on the surface of the specimens were well formed, and the overall pore size remained within the range of the bone tissue growth. The nanoindentation system was used to examine the unannealed and annealed specimens, and it was found that the annealed specimens showed a better trend in the overall material properties, and it can be inferred that the annealing process resulted in a slight increase in material strength, and the removal of residual stresses resulted in a more stable overall material structure trend. Further, according to the analysis of the stress and strain results of the compressive strength, the annealed specimen increased the structural strength of the overall scaffold with the increase in material strength. The fatigue results show that the displacement curve of the annealed samples reached a very stable trend under repeated application of a cyclic load of 50–150 N, thus concluding that no significant structural damage occurred in the scaffold. From the above results, it was found that the scaffold design of this study not only contributed to the porosity for bone tissue growth, but also had very good support in terms of force loading, which proved the effect of the structural strength of the samples after removing residual stress.

## 4. Conclusions

A 3D critical bone defect model with a defect area of 5 mm was established in the left leg tibia of New Zealand White rabbits, and the defect area was used to optimize the replacement body structure. The composite scaffold structure was designed with a dual-material combination of Ti-6Al-4V and Hap, with a 70%-65%-60%-55%-50% porosity gradient in the Ti-6Al-4V cage. Furthermore, the brittle HAp material with biodegradable property was encapsulated inside to overcome the problem of insufficient strength when the HAp is used alone. A finite element analysis method was used to set the boundary conditions with reference to the skeletal biomechanics of rabbits, and several sets of force simulations were conducted under different structural conditions. From the final design results, the composite scaffold design is able to meet the condition of uniform force distribution and achieve the effect of protecting the HAp material by using Ti-6Al-4V material as support. Based on the contact surface between the tibial defect location and the upper and lower surfaces of the scaffold, the composite scaffold design can effectively reduce the bone damage caused by the stiffness of the Ti-6Al-4V material, and further reduce the incidence of the stress shielding effect by the force performance close to the real bone. The scaffold processed by SLM has very good integrity in forming the fine pores on the surface of the scaffold. After annealing treatment, the Young’s coefficient increased from 126.44 GPa to 131.46 GPa, the hardness increased from 3.9 GPa to 4.12 GPa, and the strain decreased from 2.27% to 1.13%. The result demonstrates that the annealing process results in a more stable structural strength, which can be applied to future clinical tibial defect repair scaffolds.

## Figures and Tables

**Table 1:** Simulation control group for FEA in mechanics.

Case Number	Scaffold Material Properties		Porosity (%)	Bone Defect Size (mm)	Mesh Elements
	Scaffold Outside	Scaffold Inside			
1	Cortical Bone	Cancellous Bone	-	5	110,297
2	Ti-6Al-4V	-	* Gradient	10	912,873
3	Ti-6Al-4V	-	50	10	2,219,942
4	Ti-6Al-4V	-	70	10	1,368,419
5	Ti-6Al-4V	Ti-6Al-4V	Gradient	5	837,930
6	Ti-6Al-4V	HAp	Gradient	5	829,178

* Gradient—the gradient pore structure was designed with 70%-65%-60%-55%-50% porosity.

**Table 2:** Mechanical properties of the materials used in this study.

Material	E (GPa)	ν	ρ (g/cm^3^)	σ_yield_ (GPa)
Cortical Bone [12]	17	0.3	1.7	--
Cancellous Bone [13]	5	0.2	1.1	--
Bone Marrow [25]	3.0 × 10^−7^	0.45	-	--
Ti-6Al-4V [26]	110	0.31	4.43	0.99
Hydroxyapatite [27]	10	0.3	3.1	0.043

**Table 3:** Optimal stress relief annealing parameters [26].

Material	Heating Rate (°C/min)	Annealing Temperature (°C)	Duration Time (min)	Cooling Method
Ti-6Al-4V	2	545	105	Air Cooling

**Table 4:** Maximum equivalent stress (MPa) and strain (%) of autologous bone graft.

Maximum	b	c	d	e	f
Equivalent Stress (MPa)	--	16.286	3.355	3.332	3.661
Equivalent Strain (%)	--	0.159	0.057	0.019	0.114
Total Deformation (mm)	0.077	--	--	--	--

**Table 5:** Maximum equivalent stress (MPa) and strain (%) for Ti-6Al-4V materials with different porosity structural arrangements.

Maximum	a	b	c	d	e	f	g	h	i
Equivalent Stress (MPa)	122.190	5.342	6.372	38.729	3.704	4.137	28.068	4.200	5.355
Equivalent Strain (%)	0.113	0.032	0.038	0.036	0.021	0.032	0.026	0.025	0.032

**Table 6:** Maximum equivalent stress (MPa) and strain (%) of pore gradient support for a single Ti-6Al-4V material.

Maximum	a	b	c
Equivalent Stress (MPa)	36.331	3.887	5.019
Equivalent Strain (%)	0.038	0.023	0.030

**Table 7:** Maximum equivalent stress (MPa) and strain (%) of gradient pore support composed of Ti-6Al-4V and HAp materials.

Maximum	a	b	c	d	e	f
Equivalent Stress (MPa)	27.862	27.862	6.331	27.862	2.6	2.833
Equivalent Strain (%)	0.065	0.026	0.065	0.065	0.052	0.028

**Table 8:** Measurement data of the pore size on the surface of the sample.

Sample Type	Measuring Range	Specimen 1 (μm)	Specimen 2 (μm)	Specimen 3 (μm)	Average (μm)
Original Specimen	Scope (a)	262.0	257.11	297.51	272.21
	Scope (b)	261.76	285.18	276.67	274.54
	Scope (c)	277.92	279.65	283.16	280.24
Annealed Specimen	Scope (a)	245.8	255.12	291.16	264.03
	Scope (b)	262.43	260.09	277.81	266.78
	Scope (c)	267.03	271.28	291.16	276.49

**Table 9:** Measurement results of the nanoindentation system for Ti-6Al-4V specimens.

-	Original Specimen		Annealed Specimen	
Results term	Average	Standard Deviation	Average	Standard Deviation
Young’s modulus (GPa)	126.44	12.94	131.46	8.53
Hardness SI unit (GPa)	3.90	0.27	4.12	0.15

**Table 10:** Detailed results of the compressive strength test.

Sample Type	Load (N)	Strain (%)				
	-	Specimen 1	Specimen 2	Specimen 3	Average	Standard Deviation
Original Specimen	50	1.94	1.32	0.99	1.42	0.48
	100	2.61	1.72	1.36	1.89	0.64
	150	3.08	2.06	1.67	2.27	0.73
Annealed Specimen	50	0.58	0.58	0.59	0.58	0.01
	100	0.87	0.89	0.90	0.89	0.02
	150	1.12	1.14	1.13	1.13	0.01

## Data Availability

Not applicable.

## References

[1] Bobyn J.D., Pilliar R.M., Cameron H.U., Weatherly G.C. (1980). The optimum pore size for the fixation of porous-surfaced metal implants by the ingrowth of bone. Clin. Orthop. Relat. Res..

[2] Taniguchi N., Fujibayashi S., Takemoto M., Sasaki K., Otsuki B., Nakamura T., Matsushita T., Kokubo T., Matsuda S. (2016). Effect of pore size on bone ingrowth into porous titanium implants fabricated by additive manufacturing: An in vivo experiment. Mater. Sci. Eng. C.

[3] Schmitz J.P., Hollinger J.O. (1986). The critical size defect as an experimental model for craniomandibulofacial nonunions. Clin. Orthop. Relat. Res..

[4] Loi F., Córdova L.A., Pajarinen J., Lin T.-H., Yao Z., Goodman S.B. (2016). Inflammation, fracture and bone repair. Bone.

[5] Iulian N. (2013). Bone Grafts: Procedures, Complications and Alternatives.

[6] Arrington E.D., Smith W.J., Chambers H.G., Bucknell A.L., Davino N.A. (1996). Complications of Iliac Crest Bone Graft Harvesting. Clin. Orthop. Relat. Res..

[7] Moore W.R., Graves S.E., Bain G.I. (2001). Synthetic bone graft substitutes. ANZ J. Surg..

[8] Vecchio K.S., Zhang X., Massie J.B., Wang M., Kim C.W. (2007). Conversion of bulk seashells to biocompatible hydroxyapatite for bone implants. Acta Biomater..

[9] Best S.M., Porter A.E., Thian E.S., Huang J. (2008). Huang, Bioceramics: Past, present and for the future. J. Eur. Ceram. Soc..

[10] Zhang H., Guo Q., Liu S., Guo C., Gao Q., Tang M. (2019). Comparison of mid-term outcomes of posterior or postero-anterior approach using different bone grafting in children with lumbar tuberculosis. Medicine.

[11] Gepreel M.A.-H., Niinomi M. (2013). Biocompatibility of Ti-alloys for long-term implantation. J. Mech. Behav. Biomed. Mater..

[12] Reilly D.T., Burstein A.H. (1975). The elastic and ultimate properties of compact bone tissue. J. Biomech..

[13] Hoffler C.E., Moore K.E., Kozloff K., Zysset P., Goldstein S.A. (2000). Age, gender, and bone lamellae elastic moduli. J. Orthop. Res..

[14] Krishna B.V., Bose S., Bandyopadhyay A. (2007). Low stiffness porous Ti structures for load-bearing implants. Acta Biomater..

[15] Surmeneva M.A., Surmenev R., Chudinova E.A., Koptioug A., Tkachev M.S., Gorodzha S.N., Rännar L.-E. (2017). Fabrication of multiple-layered gradient cellular metal scaffold via electron beam melting for segmental bone reconstruction. Mater. Des..

[16] Ataee A., Li Y., Fraser D., Song G., Wen C. (2018). Anisotropic Ti-6Al-4V gyroid scaffolds manufactured by electron beam melting (EBM) for bone implant applications. Mater. Des..

[17] Zhao S., Li S., Wang S., Hou W., Li Y., Zhang L., Hao Y., Yang R., Misra R., Murr L. (2018). Compressive and fatigue behavior of functionally graded Ti-6Al-4V meshes fabricated by electron beam melting. Acta Mater..

[18] Pobloth A.-M., Checa S., Razi H., Petersen A., Weaver J.C., Schmidt-Bleek K., Windolf M., Tatai A., Roth C.P., Schaser K.-D. (2018). Mechanobiologically optimized 3D titanium-mesh scaffolds enhance bone regeneration in critical segmental defects in sheep. Sci. Transl. Med..

[19] Singh G., Sharma N., Kumar D., Hegab H. (2020). Design, development and tribological characterization of Ti–6Al–4V/hydroxyapatite composite for bio-implant applications. Mater. Chem. Phys..

[20] Fousová M., Vojtěch D., Kubásek J., Jablonská E., Fojt J. (2017). Promising characteristics of gradient porosity Ti-6Al-4V alloy prepared by SLM process. J. Mech. Behav. Biomed. Mater..

[21] Mercelis P., Kruth J. (2006). Residual stresses in selective laser sintering and selective laser melting. Rapid Prototyp. J..

[22] Schmidt F.F., Wood R.A. (1966). Heat Treatment of Titanium and Titanium Alloys.

[23] Ettefagh A.H., Zeng C., Guo S., Raush J. (2019). Corrosion behavior of additively manufactured Ti-6Al-4V parts and the effect of post annealing. Addit. Manuf..

[24] Uhthoff H.K., Poitras P., Backman D. (2006). Internal plate fixation of fractures: Short history and recent developments. J. Orthop. Sci..

[25] Tarapoom W., Puttapitukporn T. (2016). Stress Distribution in Human Tibia Bones using Finite Element Analysis. Eng. J..

[26] Pan C.-T., Lin C.-H., Huang Y.-K., Jang J., Lin H.-K., Kuo C.-N., Lin D.-Y., Huang J. (2021). Design of Customize Interbody Fusion Cages of Ti64ELI with Gradient Porosity by Selective Laser Melting Process. Micromachines.

[27] Kutz M. (2003). Standard Handbook of Biomedical Engineering & Design.

[28] Gushue D.L., Houck J., Lerner A.L. (2005). Rabbit knee joint biomechanics: Motion analysis and modeling of forces during hopping. J. Orthop. Res..

[29] Pérez M., Moreo P., García-Aznar J., Doblaré M. (2008). Computational simulation of dental implant osseointegration through resonance frequency analysis. J. Biomech..

[30] Bruni S., Martinesi M., Stio M., Treves C., Bacci T., Borgioli F. (2005). Effects of surface treatment of Ti–6Al–4V titanium alloy on biocompatibility in cultured human umbilical vein endothelial cells. Acta Biomater..

